# The Role of Artificial Intelligence of Things in Achieving Sustainable Development Goals: State of the Art

**DOI:** 10.3390/s24041091

**Published:** 2024-02-07

**Authors:** Georgios Lampropoulos, Juan Garzón, Sanjay Misra, Kerstin Siakas

**Affiliations:** 1 Department of Applied Informatics, University of Macedonia, 54636 Thessaloniki, Greece; lamprop.geo@gmail.com; 2 Faculty of Engineering, Universidad Católica de Oriente, Rionegro 111321, Colombia; fgarzon@uco.edu.co; 3 Department of Applied Data Science, Institute for Energy Technology, 1777 Halden, Norway; 4 Department of Information and Electronic Engineering, International Hellenic University, 57001 Nea Moudania, Greece; ksiakas@gmail.com; 5 Department of Production—Industrial Management, University of Vaasa, 65200 Vaasa, Finland

**Keywords:** artificial intelligence of things, artificial intelligence, internet of things, sustainability, sustainable development goals

## Abstract

With the environmental and societal changes, the achievement of sustainable development goals (SDGs) and the realization of sustainability in general is now more important than ever. Through a bibliometric analysis and scientific mapping analysis, this study aims to explore and provide a review regarding the role of artificial intelligence (AI), the Internet of Things (IoT), and artificial intelligence of things (AIoT) in realizing sustainable development and achieving SDGs. AIoT can be defined as the combination of AI with IoT to create more efficient and data-driven interconnected, intelligent, and autonomous IoT systems and infrastructure that use AI methods and algorithms. The analysis involved 9182 documents from Scopus and Web of Science (WoS) from 1989 to 2022. Descriptive statistics of the related documents and the annual scientific production were explored. The most relevant and impactful authors, articles, outlets, affiliations, countries, and keywords were identified. The most popular topics and research directions throughout the years and the advancement of the field and the research focus were also examined. The study examines the results, discusses the main findings, presents open issues, and suggests new research directions. Based on the results of this study, AIoT emerged as an important contributor in ensuring sustainability and in achieving SDGs.

## 1. Introduction

Ensuring the achievement of the Sustainable Development Goals (SDGs), which were set by the United Nations as the successors of the Millennium Development Goals (MDGs), is imperative for human development, people’s well-being, and the planet’s future prosperity and sustainability [1,2]. Within SDGs, 169 targets and 17 goals were set to be met through the collaboration and mobilization of nations, countries, governments, organizations, and individuals to secure a better future [3]. Specifically, SDGs can be regarded as a network of interrelated and interconnected goals and targets in which goals are met through the accomplishment of targets [4]. Achieving SDGs is an integral part of the social movement for sustainable development, which can be defined as a holistic approach to pursuing societal, economic, and environmental development while taking the needs of the present and future generations into consideration and ensuring an inclusive society and a sustainable environment [5].

Therefore, besides the need to foster sustainable practices, to apply drastic structural changes in all societal sectors, to capitalize on the interdependencies of the SDGs, and to adopt appropriate strategies, technologies, and policies, it is also essential to take into account the interlinkages among sectors, societal actors, and countries to successfully implement measures to meet the SDGs [6,7,8]. Additionally, it is essential to apply proper conceptual and unified frameworks, new paradigms, and relevant indicators [2,9].

Furthermore, adopting and integrating technologies in societal, environmental, and industrial sectors is necessary to meet SDGs. Artificial Intelligence (AI) is one of the most impactful and promising technologies, as it can drastically influence several domains. AI is an interdisciplinary field that focuses on creating intelligent agents that can mimic human behavior and actions, simulate human intelligence to perform specific tasks effectively and autonomously, and make decisions requiring human-level intelligence without human interventions [10,11]. AI mainly aims to provide systems and processes with increased learning, communication, and reasoning capabilities, perception, rationality, adaptability, and understanding of their environment [12,13]. Several studies have already showcased the implications of AI and blockchain in technology and society and have demonstrated its key role in achieving the SDGs and targets and attaining sustainable development [14,15]. They have also highlighted the need for appropriate safety, security, transparency, and ethical standards [16,17]. The Internet of Things (IoT) is another key technology in the fulfillment of SDGs, as recent studies have also indicated [18,19]. IoT is based on interoperable communication protocols and can be characterized as a worldwide, self-configuring, self-adjusting, dynamic, and scalable network infrastructure of interconnected and interrelated systems, devices, physical objects, and services that are embedded with sensors and software [20,21,22]. Within this flexible infrastructure, information and resources are shared between “things” that are seamlessly integrated into the network, have several advanced processes, and can communicate, sense, and interact with other “things” and their surrounding environment in real-time [23,24,25].

A new field of study called Artificial Intelligence of Things (AIoT) is gaining ground. In particular, it combines AI with IoT services and devices and capitalizes on cloud computing [26]. AIoT aims to create interconnected, intelligent, and autonomous IoT systems that use AI algorithms to interact and communicate with their environment and other systems, collect and analyze data, monitor processes, make autonomous decisions, and take actions in real-time. As a result, AIoT has the potential to transform and improve the efficiency of various sectors drastically, address societal and environmental challenges, and assist in achieving the SDGs through the optimization of the processes regarding the production, distribution, consumption, and reuse of renewable resources and the promotion of sustainable practices and decision-making. Due to its novelty, there still needs to be a systematic study that presents the state of the art of AIoT, its evolution over the years, and its use to achieve sustainable development.

Consequently, the aim of this study is to provide a systematic mapping and overview of the literature regarding AI, IoT, and AIoT and their use in achieving SDGs over the years through a bibliometric analysis. The main research question set to be explored was what the current state of the art is regarding the use of AIoT in achieving SDGs based on the existing literature. The remainder of the study is structured as follows: Section 2 goes over the method adopted and the tools used, and Section 3 presents and analyzes the results in detail. Section 4 provides a cohesive discussion about the use of AI, IoT, and AIoT to achieve SDGs, its benefits, and challenges, as well as the findings of this study. Finally, Section 5 offers conclusive remarks and suggests future research directions.

## 2. Method

One of the most widely used research methodologies to examine a broad topic and to analyze its evolution over the years is through the use of a bibliometric analysis [27]. The present study followed the guidelines presented in Donthu et al. [28] and adopted the bibliometric methodological approach showcased in Aria et al. [29]. Scopus and Web of Science (WoS) which are two accurate, relevant, and impactful databases [30,31] were used to meet the specific requirements of conducting a bibliometric study [28,32].

**Figure 1:**
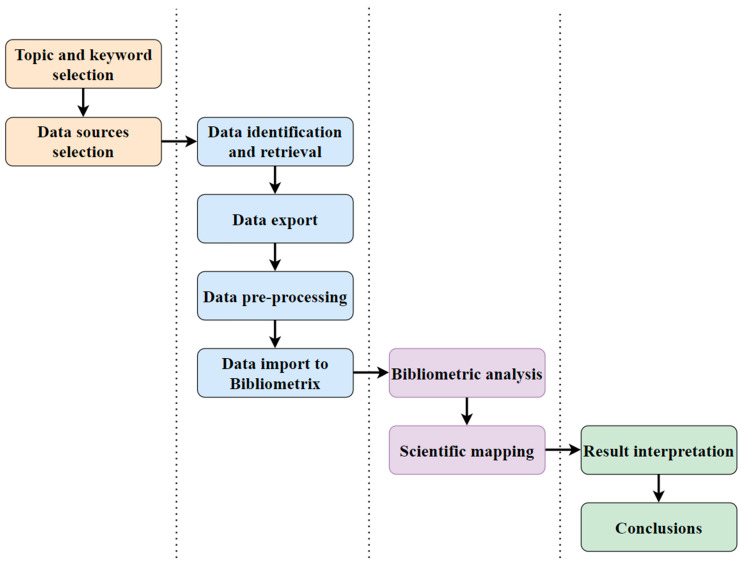
The stages of the research process followed.

The open-source R package “Bibliometrics” [29] is capable of using both Scopus and WoS data, among others, and was developed with the aim of assisting in carrying out studies that focus on exploring the literature through bibliometric analysis and scientific mapping. The query used in both databases was: (“artificial intelligence” OR “ai” OR “internet of things” OR “iot” OR “artificial intelligence of things” OR “aiot”) AND (“sustainability” OR “sustainable development” OR “sustainable development goal” OR “sdg”). All entries before 2023 were identified and retrieved. Hence, a total of 12,675 documents (8733 from Scopus and 3942 from WoS) during the period 1989–2022 were set to be examined. In total, 3208 duplicates were identified between the two datasets retrieved, which were removed. Due to the nature of the study, which is to present the current state of the art, the inclusion criteria set were for the document to involve the use of AI, IoT, and/or AIoT and focus on SDGs. Additionally, 285 documents were missing multiple key fields and were removed. As a result, the total number of documents that were in line with the inclusion criteria set and examined in this study was 9182. The result analysis, which is presented in the next section, was grouped into (1) Main information, (2) Citations, (3) Sources, (4) Authors, (5) Countries, and (6) Documents. The results are presented using tables, figures, and diagrams. The complete research process and steps followed are presented in Figure 1. Remarkably, the research process consisted of four main stages and involved (i) the initial search for appropriate topics, keywords, and data sources, (ii) the data identification, exportation, preprocessing, and import to Bibliometrix, (iii) the conduct of the bibliometric analysis and scientific mapping of the literature, and (iv) the result interpretation and conclusions.

## 3. Result Analysis

This section presents the results of the bibliometric and scientific mapping study. Particularly, the results are separated into the following categories: main information, citations, sources, authors, countries, and documents.

### 3.1. Main Information

The descriptive statistics of the studies analyzed are showcased in Table 1, which shows the description of each item and its corresponding result. Although the first document was published in 1989, the documents’ average age is 4.07, and the annual growth rate during the period from 1989–2022 was 26.34%. These facts highlight the significance of this topic throughout the years, but more so during the last 4 years. A total of 9182 documents, which were published in 3641 sources, were analyzed. Most documents were published as conference papers (3955), followed closely by documents that were published as articles in scientific journals (3776). Each record received an average of 12.92 citations. In all documents, 354,308 references and 33,505 keywords were used. In total, 23,917 authors contributed to the documents analyzed. Despite the average number of co-authors per document being 3.77 and 1111 of the documents being single-authored, the international co-authorship rate is 2.69%.

### 3.2. Citations

**Figure 2:**
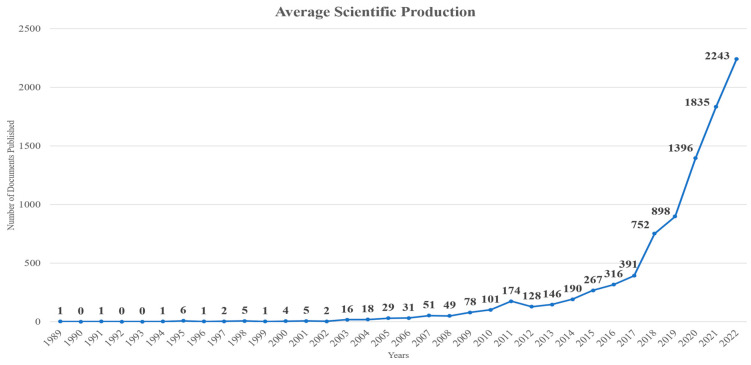
Annual scientific production based on the number of related published documents in each year.

**Figure 3:**
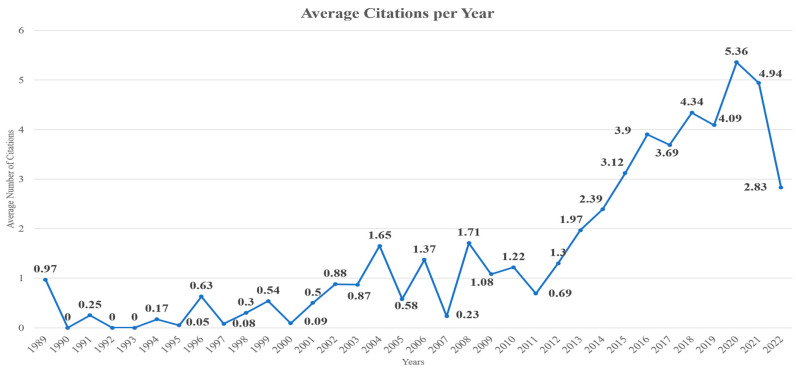
The average number of citations received of the documents published in each year.

**Figure 4:**
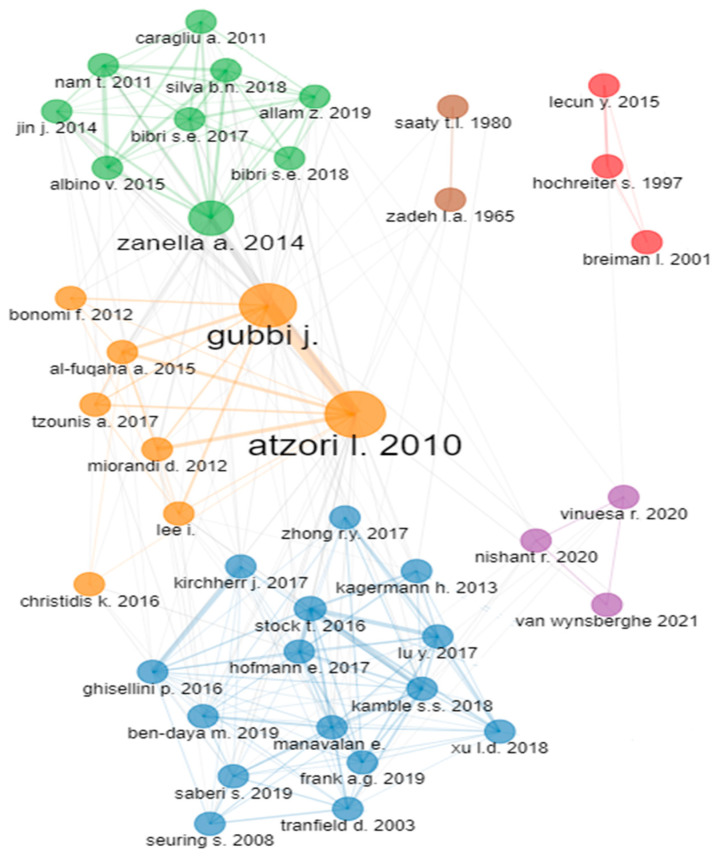
The co-citation network among the documents contained within the document collection analyzed.

The relevance and significance of ensuring sustainable development and meeting the SDGs have led to a positive annual growth rate of documents, with the number of published documents (Figure 2) and average citations per year (Figure 3) increasing annually. Particularly, in Figure 2, the X-axis refers to the years while the Y-axis represents the number of articles, while in Figure 3, the X-axis refers to the years while the Y-axis represents the number of citations. As can be seen, there is a clear increase in the number of articles examining this topic as well as the annual scientific production. Table 2 presents the documents published each year, the mean total citations per document, the mean total citations per year, and the citation years for each document during the period 2000–2022. The majority of documents were published in the last 5 years (2018–2022), with 2022 being the year in which the most documents were published (2243). Similarly, the documents from 2018 to 2021 were the ones receiving the most citations per year, with impactful documents being published yearly, as can be seen from the average total citation count per document for each year. In Figure 4, the document co-citation network is presented in which six main clusters of documents can be observed. This fact highlights the interdisciplinary nature, flexibility, and outreach of the topic.

### 3.3. Sources

**Figure 5:**
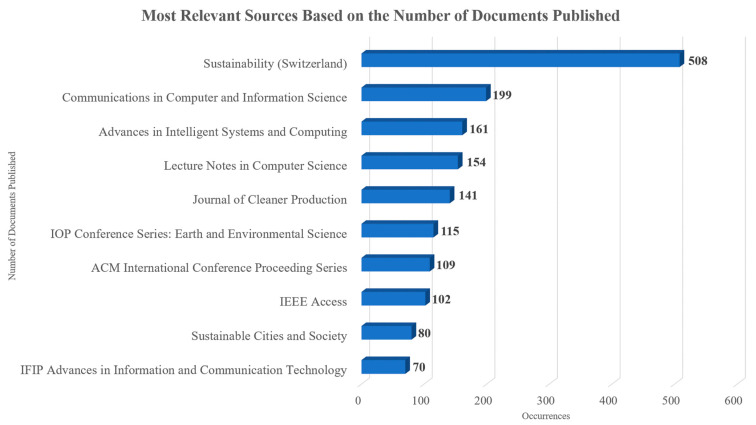
Top-10 sources based on the total number of related published documents.

**Figure 6:**
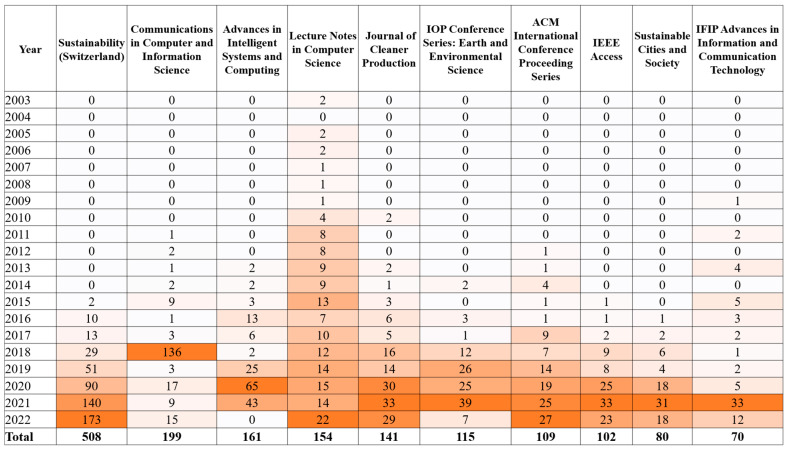
Top 10 sources production over time based on Bradford’s law for each year.

A total of 3641 sources have been used to publish documents related to the topic since 1989. The top 10 sources based on their total number of related to the topic documents published are presented in Figure 5, with “Sustainability” having the most documents published, followed by “Communications in Computer and Information Science”, “Advances in Intelligent Systems and Computing”, “Lecture Notes in Computer Science”, and “Journal of Cleaner Production”. Particularly, the X axis refers to the number of documents, while the Y axis represents the sources of the documents. As can be observed, there is a variety of sources spanning journals, conferences, and books. “Journal of Cleaner Production”, “Sustainability”, “Sustainable Cities and Society”, “IEEE Access”, and “Sensors” were the top 5 sources with the largest local impact, having the highest h-index and the total citations, while the top 10 sources based on these aspects are presented in Table 3. Mainly, in Table 3, the h_index, g_index, m_index, total citations, number of publications, and the year of the first related publication of each source are presented. The breadth of the topic and its importance become more evident when taking into account the fact that journals, conferences, and book series are among the top sources. Following Bradford’s law, three clusters emerged. The first cluster had 56 sources and 3028 published documents, the second cluster had 770 sources in which 3120 documents were published, and the third cluster had 2814 sources and 33,024 published documents. The production over time of the top 10 sources of the first cluster is presented in Figure 6. Specifically, Figure 6 presents the number of documents published in each of the top sources in each year, as well as the total number of published documents. Additionally, the color scale showcases the years that had the most published documents in each source.

### 3.4. Authors

**Figure 7:**
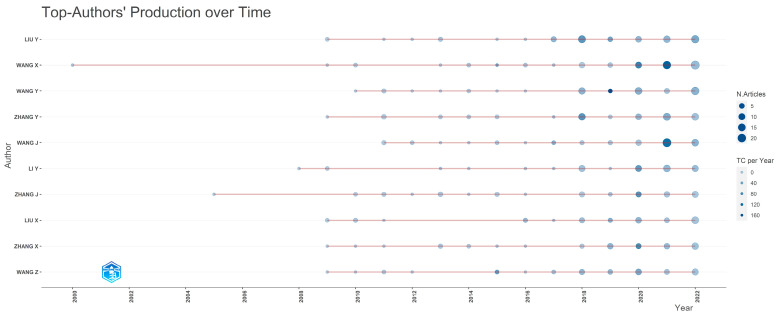
Top 10 authors’ production over time based on their annual number of published documents.

**Figure 8:**
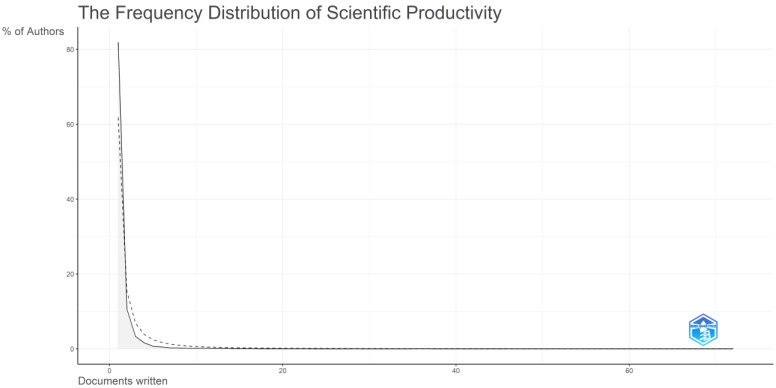
Authors’ overall productivity through Lotka’s law based on the number of documents written.

As the topic explored is multidisciplinary, authors from various expertise and backgrounds have collaborated and examined how the use of AI, IoT, and AIoT can help achieve sustainable development goals and attain sustainable development. In Table 4, which describes the author, the number of documents published on this topic, and the articles fractionalized, the top authors based on the number of related documents published are presented. Their productivity over the years is depicted in Figure 7. Particularly, the X axis of Figure 7 presents refers to the years while the Y axis represents the authors. It can be said that the top authors, according to the number of documents published, mostly started examining this topic around 2009. The top five authors that published the most were Liu Y., Wang X., Wang Y., Zhang Y., and Wang J. Figure 8 presents the authors’ productivity through Lotka’s law. Particularly, the X axis of Figure 8 represents the number of documents written while the Y axis refers to the percentage of authors. Based on the results, it can be said that the overwhelming majority of authors (81.9%) participated in the creation of a single document, followed by authors (10.6%) who participated in the development of two documents.

Furthermore, Table 5 presents the most impactful authors based on their h-index on this topic, while Table 6 showcases the most impactful authors according to the total number of citations that their work on this topic has received. Both tables showcase the author, the index, the total number of citations, the number of published documents, and the year that the first document was published. Liu Y., Wang X., Liu X., Wang J., and Zhang Y. were the top five most impactful authors based on their h-index, while Roy A., Agrawal M., Saxena N., Hossain M., and Islam S. were the top five most impactful authors when taking their total number of citations into account.

**Figure 9:**
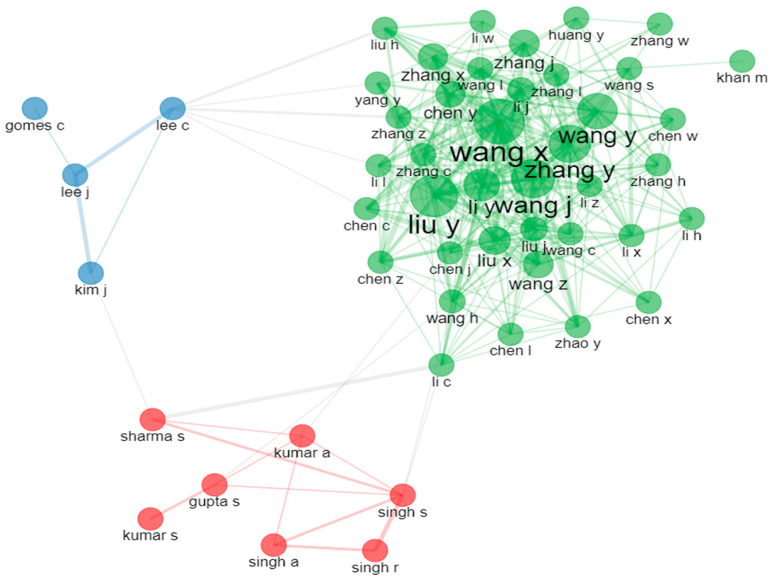
The authors’ collaboration network based on the documents contained within the document collection analyzed.

**Figure 10:**
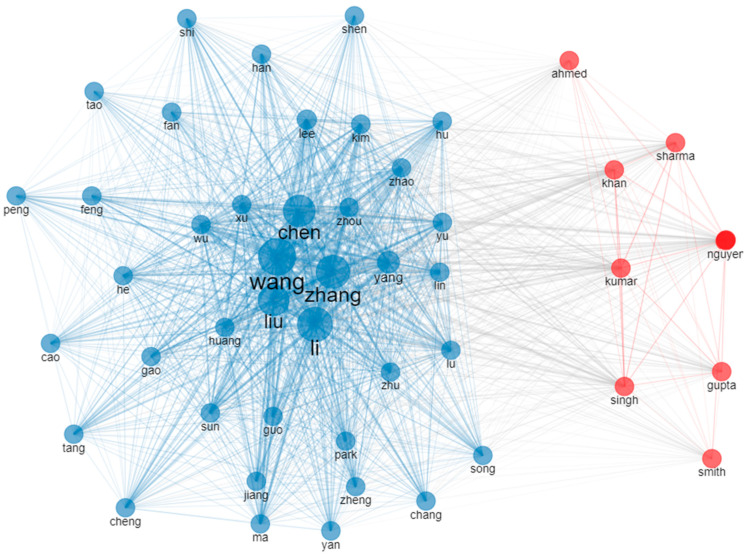
The authors’ co-citation network based on the documents contained within the document collection analyzed.

Despite the international co-authorship rate being 2.69% and the fact that 1111 documents are single-authored, an average of 3.77 authors contributed to each document. Figure 9 depicts the authors’ collaboration network, in which three main clusters can be observed, demonstrating the closest collaborators and the groups of authors mostly exploring this topic. Figure 10 showcases the authors’ co-citation network, in which five prominent authors can be observed.

**Figure 11:**
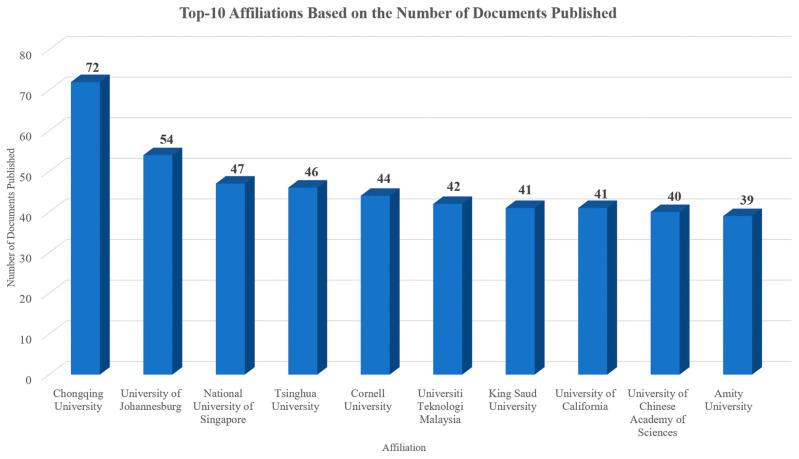
Top 10 affiliations according to the overall quantity of documents published.

**Figure 12:**
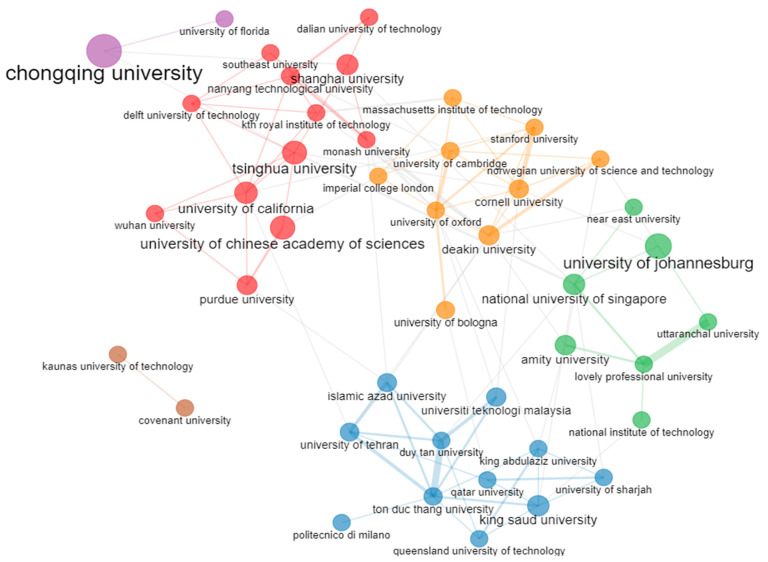
The collaboration network among the affiliations of the documents contained within the document collection analyzed.

In total, authors from 9461 different affiliations contributed to documents of the collection analyzed. The most relevant affiliations based on the number of studies which were conducted on this topic are presented in Figure 11. Specifically, the X axis of the figure refers to the number of documents, while the Y axis represents the affiliations of the authors. As can be seen, the top affiliations all have at least 39 documents published on this topic. The affiliations’ collaboration network is showcased in Figure 12, in which six clusters can be observed, a fact that highlights the flexibility, broadness, and interdisciplinary nature of this topic. Chongqing University, University of Johannesburg, National University of Singapore, Tsinghua University, and Cornell University were the top five sources with the largest number of related to the topic published documents.

### 3.5. Countries

**Figure 13:**
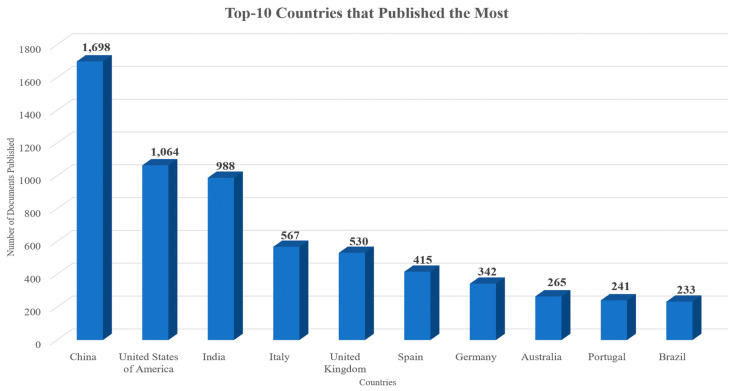
Top 10 countries whose authors published the most documents throughout the years examined.

**Figure 14:**
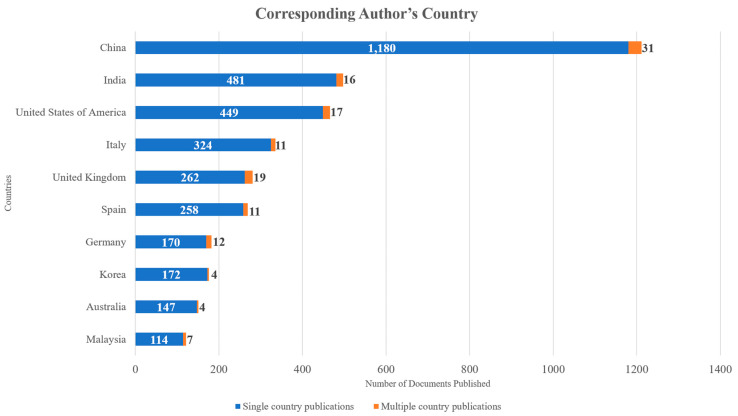
Top 10 countries whose authors published the most documents throughout the years examined based on corresponding author’s country.

**Figure 15:**
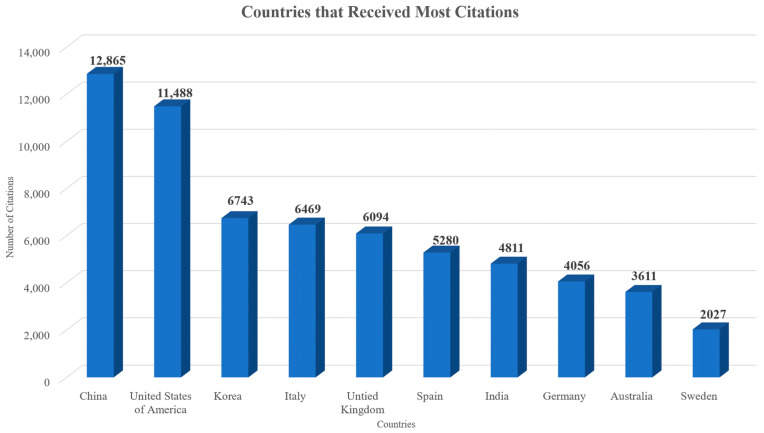
Top 10 countries whose published documents on the topic received most citations over the years.

**Figure 16:**
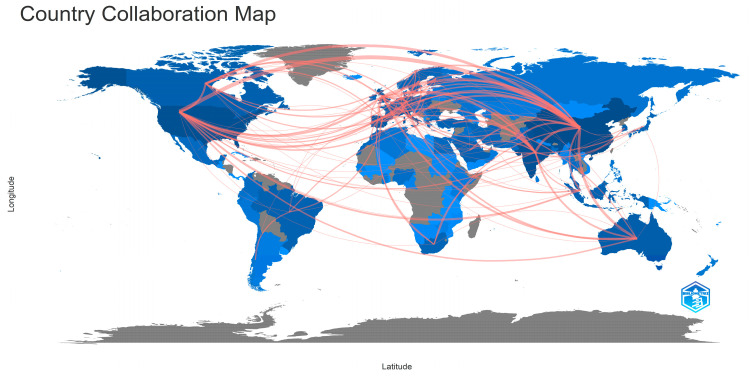
Mapping of the countries which collaborated in the publication of documents on this topic.

Authors from a total of 117 countries contributed to the documents published on this topic. Each country’s scientific production, when all authors’ nationalities are considered, is presented in Figure 13. The countries that published the most, according to the corresponding author’s country, are depicted in Figure 14. In both figures, the X axis represents the countries while the Y axis refers to the number of published documents. It is worth highlighting the drastic changes to the number of documents published, even among the top countries. Additionally, in both cases, China, the United States of America, India, Italy, and the United Kingdom were the countries that contributed to the publication of the most documents related to the topic. Figure 15 showcases the countries whose published documents received the most citations. In Figure 15, the X axis represents the countries, while the Y axis refers to the total number of citations received. It is worth highlighting the drastic changes to the number of citations, even among the top countries, based on the number of articles published. The top five most cited countries were China, the United States of America, Korea, Italy, and the United Kingdom. The country collaboration map is depicted in Figure 16, which highlights the global significance of the specific topic.

### 3.6. Documents

**Figure 17:**
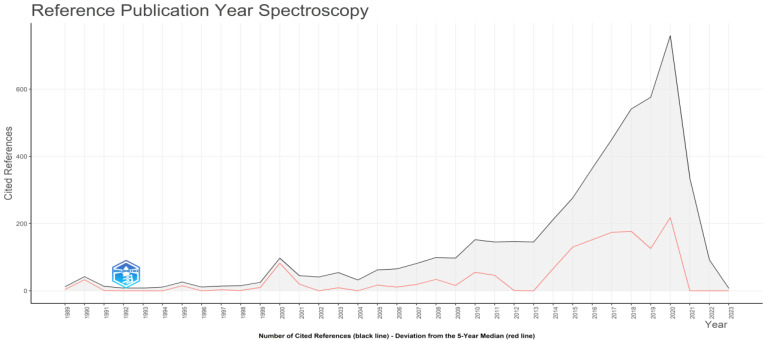
Reference publication year spectroscopy according to the cited references in each year.

The top 15 most cited documents of the 9182 documents included in this study are presented in Table 7. Table 7, in particular, describes the related reference, DOI, the total number of citations it received, the total number of citations per year, and the normalized total number of citations. The top five most impactful documents, according to the total citation number, were the ones published by Agrawal et al. [33], Riazul Islam et al. [34], Kshetri [35], Kusiak [36], and Kamble et al. [37]. It must be noted that the total number of citations received is taken into account when analyzing the most impactful documents. Hence, survey and literature review articles generally have more citations than documents of technical content. Despite this fact, both theoretical and practical documents are required, and both contribute significantly to shaping this field of study. The importance of these publications can also be detected in Figure 17, which depicts the reference publication year spectroscopy diagram with its X-axis referring to the years and its Y-axis referring to the number of cited references.

**Figure 18:**
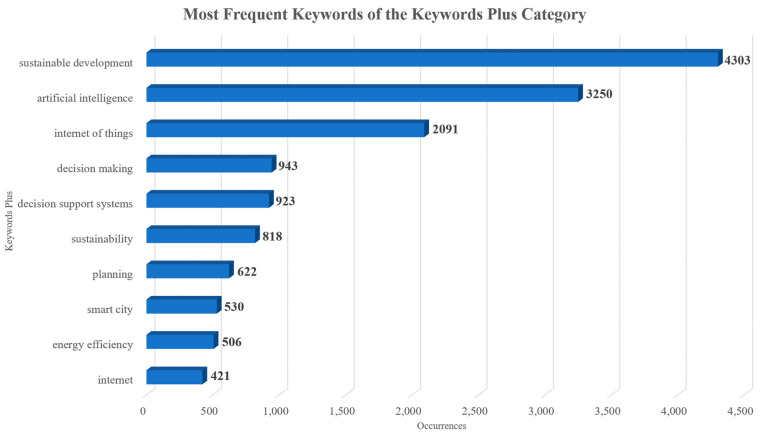
The top 10 most commonly used keywords of the keywords plus category within the published documents of this collection.

**Figure 19:**
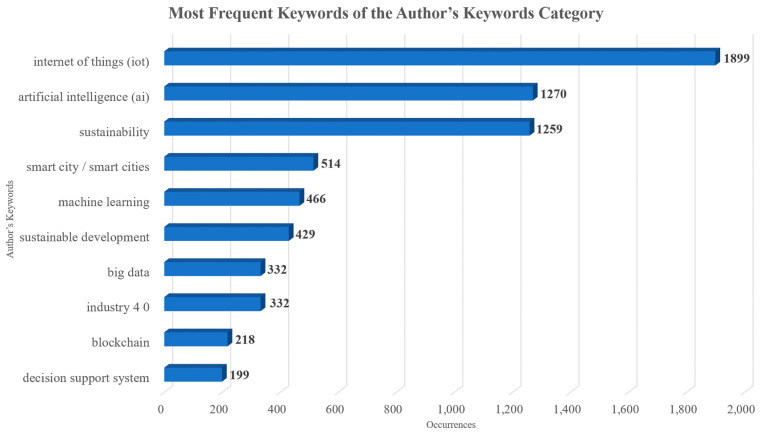
The top 10 most commonly used keywords of the author’s keywords category within the published documents of this collection.

**Figure 20:**
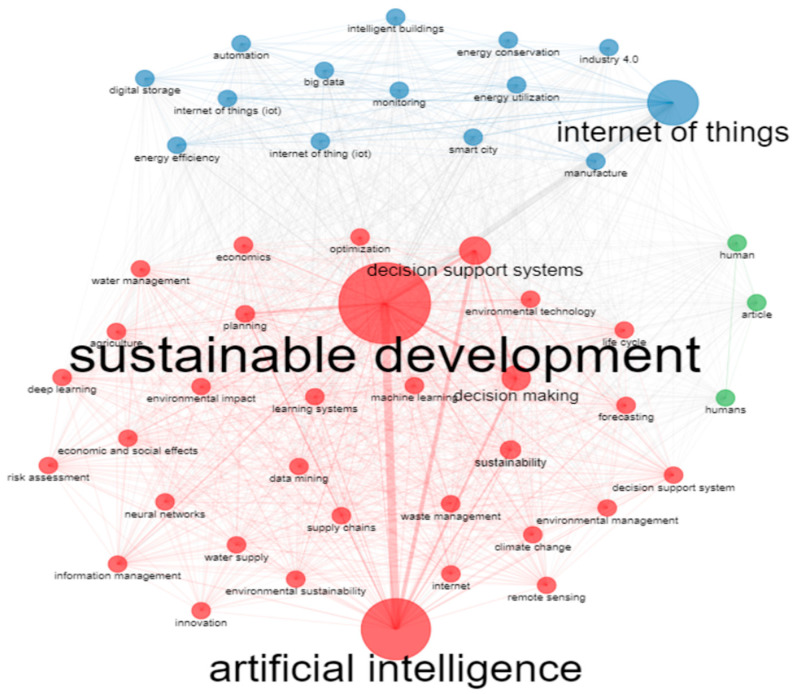
Co-occurrence network of the keywords of the keyword plus category within the documents of the collection.

**Figure 21:**
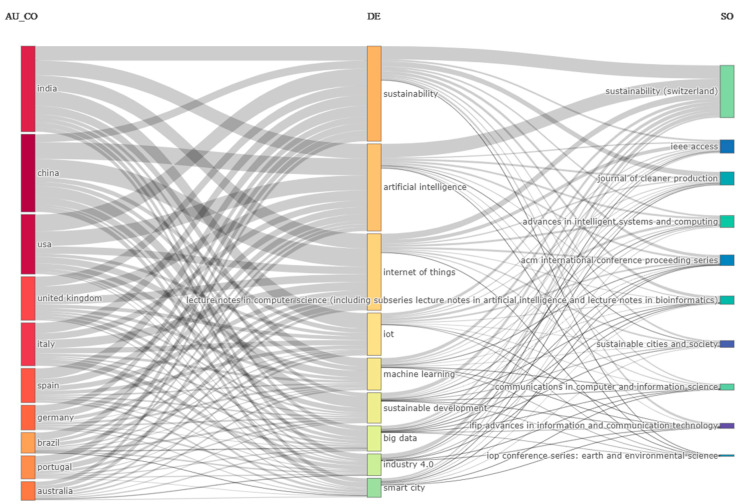
The relationship among the top 10 countries with the most published documents, most common keywords, and sources with the most published documents.

As far as the keywords are concerned, although both author’s keywords and keywords plus can adequately present the document knowledge structure when using data from both Scopus and WoS, the use of keywords plus generally presents a more cohesive representation [47]. Hence, unless specified, the term “keywords” in the text will refer to the use of keywords plus. The topmost commonly used keywords were “sustainable development”, “artificial intelligence”, “internet of things”, “decision making”, and “decision support systems” and their frequency is presented in Figure 18. The topmost common author’s keywords were “Internet of Things (IoT)”, “artificial intelligence”, “sustainability”, “smart city/smart cities”, and “machine learning” and their frequency is displayed in Figure 19. Both figures present the frequency in their X-axis and the related keywords in their Y-axis. Based on the results, the diverse nature of the topic is highlighted. Furthermore, the co-occurrence network of the keywords used within the documents examined is showcased in Figure 20, in which three main clusters of keywords can be seen. The relationships between the top 10 most productive countries, most common keywords, and most frequent sources are presented in Figure 21.

**Figure 22:**
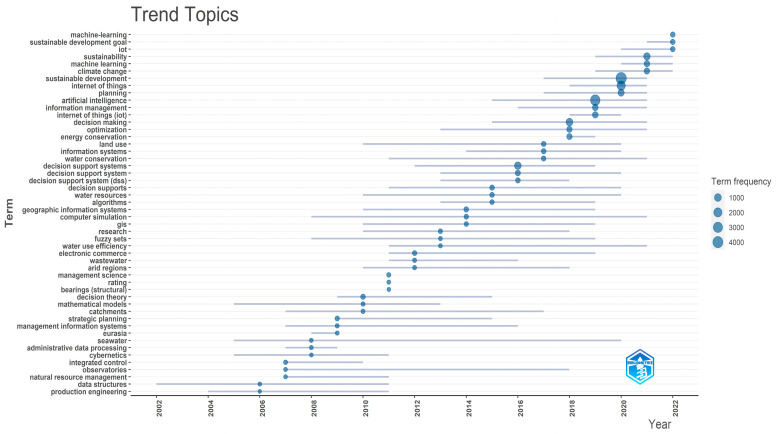
The evolution of the topic trends throughout the years based on the frequency of keywords of the category keyword plus used.

**Figure 23:**
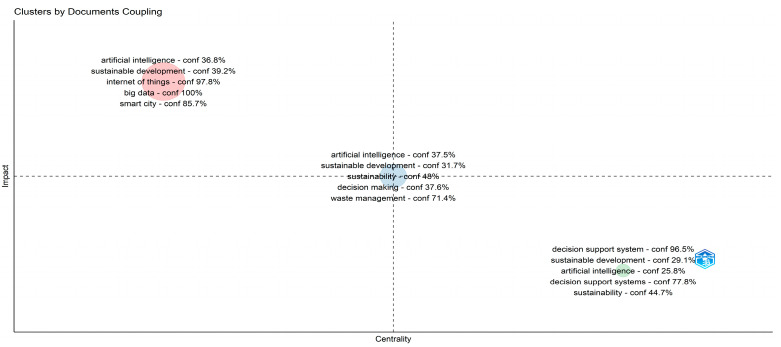
Mapping of the published documents using clustered by coupling and keywords of the keywords plus category as representations of each cluster.

**Figure 24:**
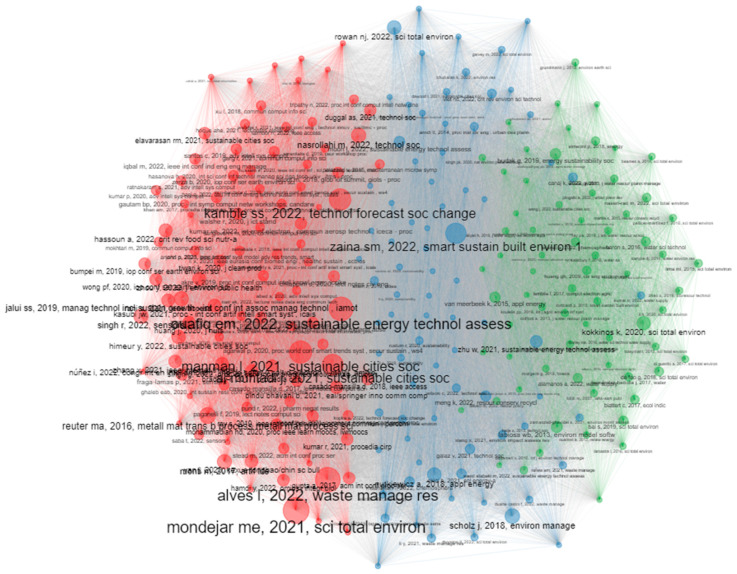
Network of documents clustered by coupling.

The topic trends from 2002 to 2022, which are based on keywords plus, are displayed in Figure 22, with the X axis referring to the years and the Y axis presenting the topic trends based on the related keywords. The initial focus on the infrastructure and digital technologies, their steady integration into several domains that influence sustainability, and the shift of interest in climate change, environment protection, and sustainable development goals over the last years can be observed. Global citation score as an impact measure and coupling measured by keywords were used to cluster the documents. In total, three clusters were created. Figure 23 presents the map of documents clustered by coupling, while Figure 24 showcases the network of documents clustered by coupling. In both figures, the three clusters emerged following the coupling of documents, which can be seen.

**Figure 25:**
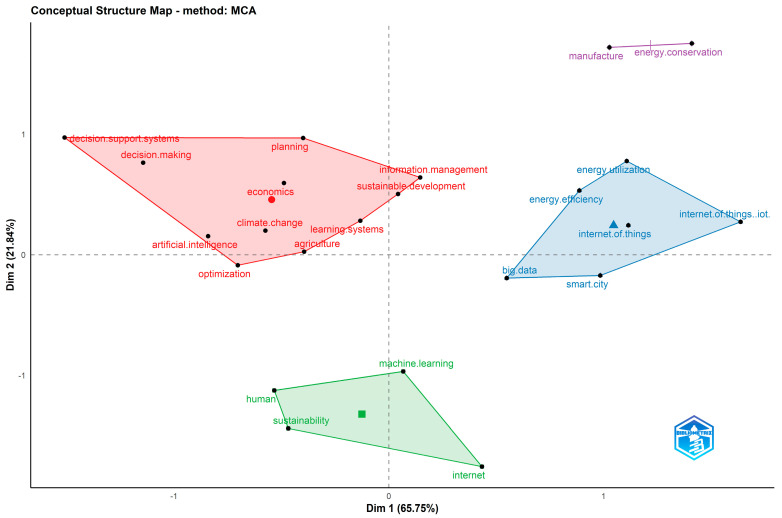
Conceptual structure map of the topic using keywords of the keywords plus category as representations.

**Figure 26:**
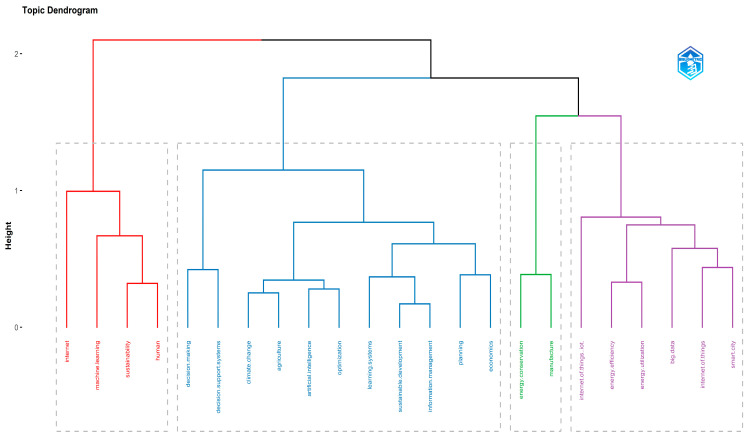
Topic dendrogram based on the frequency of keywords of the keyword plus category.

The conceptual and thematic structure of the topic were also explored. Particularly, Figure 25 depicts the topic conceptual structure map while Figure 26 presents the dendrogram of the emerged topic keywords. Within the conceptual structure map, a total of four clusters emerged. The first one involves AI and its role in supporting sustainable development through autonomous decision support and planning systems. The second one refers to the use of IoT within the context of smart cities to capitalize on interconnectivity and big data to improve energy production, distribution, and consumption. The third cluster involves the human factor, the role of the Internet and communication, and the use of machine learning to improve sustainability. Lastly, the fourth cluster refers to the integration of AI and IoT in the manufacturing, industrial, and energy sectors.

**Figure 27:**
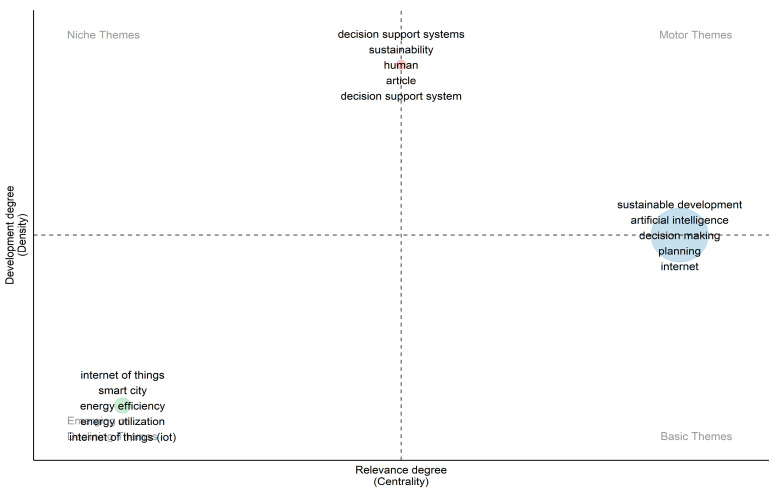
Thematic map of the topic following their development and relevance degrees.

**Figure 28:**
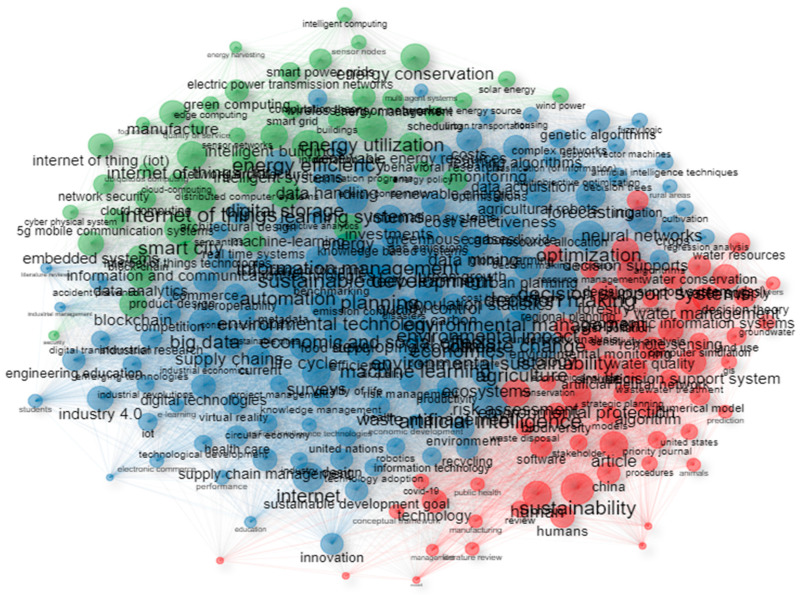
Thematic network of the topic based on the keywords of the keyword plus category.

**Figure 29:**
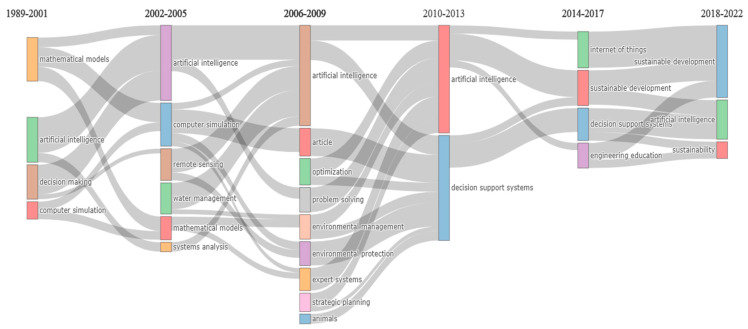
Thematic evolution of the topic based on the keywords of the keyword plus category in four-year intervals.

In Figure 27 and Figure 28, the three themes that emerged from clustering the keywords of the documents are showcased. One was related to the use of AI, the second one to the use of IoT, and the third one to the human factors and the use of decision support systems. Furthermore, since the documents published were from the period 1989–2022, six time periods were set to explore the thematic evolution of the topic, which can be seen in Figure 29. The periods were divided into (i) 1989–2001, (ii) 2002–2005, (iii) 2006–2009, (iv) 2010–2013, (v) 2014–2017, and (vi) 2018–2022. The use of AI is profound in each time period, while IoT started to appear from 2014 to 2017 and afterward. The initial focus on the technologies and then on specific domains was observed. The use of decision support systems to assist humans was also evident. Although a shift of focus toward sustainable development was observed even from 2002–2005, the main shift toward sustainability and sustainable development goals was noticed in the period 2014–2017 and afterward.

## 4. Discussion

The 17 SDGs set by the United Nations to be achieved by 2030 characterize a global partnership among all countries to share a commonly accepted plan for meeting them and attaining sustainable development, which, in turn, will lead to dignity, peace, and prosperity. Nonetheless, to address this urgent call for action, innovative solutions are required to ensure the achievement of the SDGs. The current decade is regarded as the decade of action toward reaching the 2023 milestone. Ambitions and plans must now turn into reality. Novel technologies, such as AI, IoT, and AIoT, can contribute to facilitating and accelerating the progress toward the realization of the SDGs. The acceleration and transfer of technological innovations is a common concern of humankind, transcending the boundaries of a single country and requiring international collaboration and collective actions. In this context, digital advances are regarded as crucial for supporting and achieving each of the 17 SDGs.

This bibliometric and scientific mapping study aimed to analyze how AI, IoT, and AIoT are being used in the context of sustainable development, examine their role in achieving the SDGs, and explore their evolution over the years. To address this aim, the descriptive statistics and characteristics of the related studies, the most common keywords, the most popular topics, and the most relevant and impactful sources, authors, affiliations, countries, and articles, as well as how the topic evolved over the years, were explored. The study involved 9182 documents from Scopus and WoS published in 3641 different sources from 1989 to 2022. The results were grouped into main information, citations, sources, authors, countries, and documents.

To sum up the results of the analysis, the scientific interest regarding the use of AI, IoT, and AIoT in achieving SDGs and sustainable development has been increasing annually, with a significant increase in the annual production of documents being observed since 2018 and afterward. The annual growth rate is 26.34%, the average age of the documents is 4.07 years, and each article received an average of 12.9 citations, which highlights the recency of the topic over the last few years. Most documents were published as conference papers, followed closely by documents that were published in scientific journals. The international co-authorship rate was 2.69%, while the average number of co-authors in each document was 3.77. Most documents were published from 2018 to 2021, and the average number of citations per year increased from 2011 to 2020.

In total, 3641 international outlets were used, which were clustered into three groups following Bradford’s law. “Journal of Cleaner Production” (h-index 47), “Sustainability” (h-index 42), “Sustainable Cities and Society” (h-index 30), “IEEE Access” (h-index 24), and “Sensors” (h-index 21) were the top five sources with the largest local impact, having the highest h-index and the most total citations. When taking the sources production over time into account, “Sustainability” (508 documents), “Communications in Computer and Information Science” (199 documents), “Advances in Intelligent Systems and Computing” (161 documents), “Lecture Notes in Computer Science” (154 documents), and “Journal of Cleaner Production” (141 documents) were the top five sources.

A total of 23,917 different authors from different disciplines and backgrounds have contributed to these studies. The vast majority of authors were involved either in a single article (81.9%) or two articles at the most. Liu Y. (h-index 19), Wang X. (h-index 14), Wang Y. (h-index 13), Zhang Y. (h-index 13), and Wang J. (h-index 13) were the top five authors that published the most. Liu Y. (72 documents), Wang X. (68 documents), Liu X. (64 documents), Wang J. (63 documents), and Zhang Y. (56 documents) were the top five most impactful authors based on their h-index. Roy A. (2177 total citations), Agiwal M. (2152 total citations), Saxena N. (2152 total citations), Hossain M. (1966 total citations), and Islam S. (1854 total citations) were the top five most impactful authors when taking their total number of citations into account.

The top five affiliations that produced the most significant number of publications out of the 9461 different affiliations within this dataset were Chongqing University (72 documents), University of Johannesburg (54 documents), National University of Singapore (47 documents), Tsinghua University (46 documents), and Cornell University (44 documents). In the studies examined, authors from 177 different countries were involved. The countries that contributed to the publication of the most documents related to the topic were China (1698 documents), the United States of America (1064 documents), India (988 documents), Italy (567 documents), and the United Kingdom (530 documents), while China (12,865 total citations), the United States of America (11,488 total citations), Korea (6743 total citations), Italy (6469 total citations), and the United Kingdom (6094 total citations) were the top five most cited countries.

Out of the 9182 documents examined, the top five most impactful ones according to the total number of citations received were Agiwal et al. [33] (total citations: 2152), Riazul Islam et al. [34] (total citations: 1849), Kshetri [35] (total citations: 865), Kusiak [36] (total citations: 654), and Kamble et al. [37] (total citations: 624). “Sustainable development”, “artificial intelligence”, “internet of things”, “decision making”, and “decision support systems” were the most commonly used keywords. Based on the co-occurrence network of the keywords, most documents were associated with sustainable development, AI, and IoT. This fact was in line with the thematic evolution of the topic.

## 5. Conclusions

This study aimed to examine the use of AI, IoT, and AIoT in the context of sustainable development, explore their role in achieving the SDGs, and analyze how they evolved over the years. Hence, this study contributed a scientific mapping analysis and a bibliometric analysis, which involved 9182 documents from Scopus and WoS over the period from 1989–2022. Different factors were taken into account to analyze the data. The descriptive statistics of the related documents and the annual scientific production were explored. The most relevant and impactful authors, articles, outlets, affiliations, countries, and keywords were identified. Moreover, the most popular topics and research directions throughout the years, the advancement of the field, and the research focus were also examined. The recency and significance of the topic are evident in the results. The increasing number of published documents on this topic in all types of sources over the last years and the fact that the topic is widely studied by researchers from different disciplines and countries across all continents from both private and public universities and institutes further showcase the importance of achieving SDGs. The gradual transition from traditional systems to technology-enabled intelligent systems and the focus shifting to pursuing more sustainable approaches, methods, and resources were observed. AIoT emerged as an important aspect of realizing sustainability and meeting the SDGs.

Consequently, the results and findings of this study contribute to bridging the gap in the existing literature regarding the adoption and integration of AI, IoT, and AIoT in the context of sustainable development. Given the fact that only seven years remain until the 2030 milestone, this study highlights the role of AI, IoT, and AIoT as significant contributors to achieving SDGs. Despite this fact, as was evident from the results, AIoT as a field is currently in its infancy but has demonstrated great potential to influence and transform several sectors and be a leading aspect in achieving a sustainable future. Given the importance of creating ideal conditions that will enable sustainable development and the achievement of SDGs, this study hopes to pave the way for new lines of work to be developed.

Due to the interdisciplinary nature of SDGs and AIoT, future studies should focus on exploring their intersection from different directions while targeting at specific SDGs or domains. Thus, collaboration among researchers of different backgrounds, expertise, and disciplines is encouraged. There is also a clear need for common evaluation metrics, standards, and models to be created. Ensuring the security and safety of critical infrastructure in the context of SDGs is also crucial. Hence, future studies should examine how AIoT can be used to enhance the security of critical infrastructure. Finally, there currently needs to be more empirical studies that involve the application of AIoT-enabled systems and platforms in real scenarios.

## Figures and Tables

**Table 1:** Main information of the document collection.

Description	Results
Timespan	1989:2022
Sources (journals, books, etc.)	3641
Documents	9182
Annual growth rate %	26.34
Document average age	4.07
Average citations per doc	12.92
References	354,308
DOCUMENT CONTENTS	
Keywords plus (ID)	33,505
Author’s keywords (DE)	20,809
AUTHORS	
Authors	23,917
Authors of single-authored docs	982
AUTHORS COLLABORATION	
Single-authored docs	1111
Co-authors per doc	3.77
International co-authorships %	2.69

**Table 2:** Main information about the documents published in each year, their mean total and yearly citations, and citable years.

Year	N	MeanTCperArt	MeanTCperYear	CitableYears
2000	4	2	0.09	23
2001	5	11	0.5	22
2002	2	18.5	0.88	21
2003	16	17.31	0.87	20
2004	18	31.44	1.65	19
2005	29	10.38	0.58	18
2006	31	23.29	1.37	17
2007	51	3.63	0.23	16
2008	49	25.61	1.71	15
2009	78	15.13	1.08	14
2010	101	15.88	1.22	13
2011	174	8.26	0.69	12
2012	128	14.33	1.3	11
2013	146	19.7	1.97	10
2014	190	21.49	2.39	9
2015	267	24.95	3.12	8
2016	316	27.27	3.9	7
2017	391	22.12	3.69	6
2018	752	21.68	4.34	5
2019	898	16.34	4.09	4
2020	1396	16.09	5.36	3
2021	1835	9.88	4.94	2
2022	2243	2.83	2.83	1

**Table 3:** Top 10 most impactful sources based on the h-index metric.

Source	h_index	g_index	m_index	TC	NP	PY_start
Journal of Cleaner Production	47	75	3.357	6401	141	2010
Sustainability (Switzerland)	42	73	4.667	8641	508	2015
Sustainable Cities and Society	30	49	3.75	2683	80	2016
IEEE Access	24	63	2.667	4116	102	2015
Sensors (Switzerland)	21	33	1.909	1391	33	2013
Science of the Total Environment	19	34	1.583	1206	36	2012
IEEE Internet of Things Journal	17	35	1.889	1272	35	2015
Technological Forecasting and Social Change	17	25	1.889	1421	25	2015
Energies	16	32	1.333	1161	65	2012
International Journal of Production Research	15	22	1.364	1510	22	2013

**Table 4:** Top 10 authors according to the number of their published documents in this collection.

Authors	Articles	Articles Fractionalized
Liu Y	72	19.02
Wang X	68	15.91
Wang Y	64	21.21
Zhang Y	63	19.47
Wang J	56	14.94
Li Y	50	11.88
Zhang J	48	12.33
Liu X	45	13.18
Zhang X	44	12.38
Wang Z	43	12.36

**Table 5:** Most impactful authors based on their h-index on this topic.

Author	h_index	g_index	m_index	TC	NP	PY_start
Liu Y	19	33	1.267	1190	72	2009
Wang X	14	36	0.583	1341	68	2000
Liu X	13	25	0.867	658	45	2009
Wang J	13	25	1	666	56	2011
Zhang Y	13	27	0.867	812	63	2009
Li Z	12	24	0.923	588	31	2011
Singh S	12	25	1.5	648	29	2016
Khan M	11	26	0.846	679	35	2011
Li H	11	16	0.846	294	28	2011
Li Y	11	23	0.688	571	50	2008
Wang Y	11	35	0.786	1296	64	2010
Wang Z	11	33	0.733	1118	43	2009
Zhang X	11	25	0.733	650	44	2009

**Table 6:** Most impactful authors based on the total number of citations on this topic.

Author	h_index	g_index	m_index	TC	NP	PY_start
Roy A	2	3	0.25	2177	3	2016
Agiwal M	1	1	0.125	2152	1	2016
Saxena N	1	1	0.125	2152	1	2016
Hossain M	7	8	0.538	1966	8	2011
Islam S	2	3	0.2	1854	3	2014
Kabir M	1	1	0.111	1849	1	2015
Kwak D	1	1	0.111	1849	1	2015
Kwak K	1	1	0.111	1849	1	2015
Müller J	6	6	0.857	1363	6	2017
Wang X	14	36	0.583	1341	68	2000

**Table 7:** Top 10 most cited documents based on the total number of citations received.

Reference	DOI	Total Citations	TC per Year	Normalized TC
Agiwal et al. [33]	10.1109/COMST.2016.2532458	2152	269	78.91
Riazul Islam et al. [34]	10.1109/ACCESS.2015.2437951	1849	205.44	74.12
Kshetri [35]	10.1016/j.ijinfomgt.2017.12.005	865	144.17	39.91
Kusiak [36]	10.1080/00207543.2017.1351644	654	109	30.17
Kamble et al. [37]	10.1016/j.psep.2018.05.009	624	104	28.79
Wang et al. [38]	10.1109/TSG.2018.2818167	602	120.4	36.83
Shrouf et al. [39]	10.1109/IEEM.2014.7058728	601	60.1	27.97
Deb et al. [40]	10.1016/j.rser.2017.02.085	512	73.14	23.14
Vinuesa et al. [17]	10.1038/s41467-019-14108-y	501	125.25	31.13
Müller et al. [41]	10.3390/su10010247	492	82	22.7
Nourani et al. [42]	10.1016/j.jhydrol.2014.03.057	475	47.5	22.1
Niu et al. [43]	10.1038/ncomms9975	460	51.11	18.44
Manavalan and Jayakrishna [44]	10.1016/j.cie.2018.11.030	427	85.4	26.13
Jiang and Wen [45]	10.1108/IJCHM-03-2020-0237	402	100.5	24.98
Allam and Dhunny [46]	10.1016/j.cities.2019.01.032	398	79.6	24.35

## Data Availability

The data analyzed in this study are available from the corresponding author on reasonable request.
